# The Neuroprotective Effects of the CB2 Agonist GW842166x in the 6-OHDA Mouse Model of Parkinson’s Disease

**DOI:** 10.3390/cells10123548

**Published:** 2021-12-16

**Authors:** Hao Yu, Xiaojie Liu, Bixuan Chen, Casey R. Vickstrom, Vladislav Friedman, Thomas J. Kelly, Xiaowen Bai, Li Zhao, Cecilia J. Hillard, Qing-Song Liu

**Affiliations:** 1 Department of Pharmacology and Toxicology, Medical College of Wisconsin, 8701 Watertown Plank Road, Milwaukee, WI 53226, USA; haoyu@mcw.edu (H.Y.); xiaojieliu@mcw.edu (X.L.); bchen@mcw.edu (B.C.); cvickstrom@mcw.edu (C.R.V.); vfriedman@mcw.edu (V.F.); tjkelly@mcw.edu (T.J.K.); chillard@mcw.edu (C.J.H.); 2 Department of Exercise Physiology, Beijing Sport University, Beijing 100084, China; zhaolispring@126.com; 3 Department of Cell Biology, Neurobiology and Anatomy, Medical College of Wisconsin, 8701 Watertown Plank Road, Milwaukee, WI 53226, USA; xibai@mcw.edu

**Keywords:** Parkinson’s disease, 6-OHDA, GW842166x, dopamine neuron, motor function, I_h_, cannabinoid type 2 receptor, substantia nigra pars compacta, neuroprotection

## Abstract

Parkinson’s disease (PD) is a chronic neurodegenerative disorder associated with dopamine neuron loss and motor dysfunction. Neuroprotective agents that prevent dopamine neuron death hold great promise for slowing the disease’s progression. The activation of cannabinoid (CB) receptors has shown neuroprotective effects in preclinical models of neurodegenerative disease, traumatic brain injury, and stroke, and may provide neuroprotection against PD. Here, we report that the selective CB2 agonist GW842166x exerted protective effects against the 6-hydroxydopamine (6-OHDA)-induced loss of dopamine neurons and its associated motor function deficits in mice, as shown by an improvement in balance beam walking, pole, grip strength, rotarod, and amphetamine-induced rotation tests. The neuroprotective effects of GW842166x were prevented by the CB2 receptor antagonist AM630, suggesting a CB2-dependent mechanism. To investigate potential mechanisms for the neuroprotective effects of GW842166x, we performed electrophysiological recordings from substantia nigra pars compacta (SNc) dopamine neurons in ex vivo midbrain slices prepared from drug-naïve mice. We found that the bath application of GW842166x led to a decrease in action potential firing, likely due to a decrease in hyperpolarization-activated currents (I_h_) and a shift of the half-activation potential (*V*_1/2_) of I_h_ to a more hyperpolarized level. Taken together, the CB2 agonist GW842166x may reduce the vulnerability of dopamine neurons to 6-OHDA by decreasing the action potential firing of these neurons and the associated calcium load.

## 1. Introduction 

Parkinson’s disease (PD) is a progressive neurodegenerative disorder associated with motor dysfunction and neuropsychiatric symptoms, primarily characterized by the age-dependent loss of dopaminergic projections in the nigrostriatal pathway [1,2]. The classical motor symptoms of PD include bradykinesia, resting tremors, rigidity, and postural instability [2,3]. These symptoms arise from the progressive loss of dopamine neurons in the substantia nigra pars compacta (SNc) and the reduction in dopamine release in the striatum. The dopamine precursor levodopa and dopamine receptor agonists are commonly prescribed treatments for PD [4,5]. Although dopamine replacement therapies relieve symptoms of PD after their manifestation, they do not slow the continued degeneration of dopamine neurons and, ultimately, produce adverse effects such as dyskinesia and compulsive behavior that limit their therapeutic utility [6]. Thus, the identification of neuroprotective agents that can prevent or slow the death of dopamine neurons holds great promise for slowing disease progression and reducing the risk of onset in vulnerable individuals.

Emerging evidence suggests that the activation of cannabinoid (CB) receptors may contribute to neuroprotection against stroke and neurodegenerative diseases, including PD [7]. The endocannabinoid (eCB) ligand 2-arachidonoylglycerol (2-AG) is degraded primarily by monoacylglycerol lipase (MAGL), and the selective MAGL inhibitor JZL184 has exhibited neuroprotective effects in mouse models of Alzheimer’s disease [8] and PD [9]. Preclinical studies have shown that the non-selective CB1/CB2 agonists WIN-55,212-2 and HU-210 improved PD-associated deficits in motor function [10,11,12] and increased the survival of SNc dopamine neurons in multiple PD models, including the 1-methyl-4-phenyl-1,2,3,6-tetrahydropyridine (MPTP) model [10,13,14]. Randomized, double-blind, placebo-controlled clinical trials indicated that nabilone, a dual CB1/CB2 agonist, reduced dyskinesia [15,16] and painful dystonia in PD patients [17]. However, the CB1 agonism produces psychoactive effects and may pose a risk of abuse and dependence, whereas selective CB2 agonists lack psychoactivity [18]. CB2 receptors are expressed in midbrain dopamine neurons [19,20,21]. The CB2 gene expression is significantly increased in the SN of PD postmortem brain samples [22]. β-Caryophyllene (BCP), a naturally occurring selective CB2 receptor agonist, attenuated dopamine neuron loss in a rotenone-induced rat model of PD through its anti-inflammatory and antioxidant activities [23]. The selective CB2 agonists JWH-133 and AM1241 attenuated MPTP-induced degeneration of dopamine neurons and axonal terminals [23,24]. The present study was undertaken to test whether the novel CB2 agonist GW842166x has neuroprotective effects against dopamine neuron loss and its associated motor deficits in the neurotoxic 6-hydroxydopamine (6-OHDA)-induced mouse model of PD, which has been extensively used to study motor function deficits within subjects by assessing rotational behavior [25,26]. We chose GW842166x because it was found to be safe and well-tolerated with an established bioavailability and no serious adverse effects in a phase two clinical trial for pain relief [27]. Mice first received unilateral injections of 6-OHDA or vehicle control into the dorsal striatum and then received chronic treatments with GW842166x by daily systemic injections for 3 weeks. Dopamine neuron loss was confirmed by the immunohistochemistry of tyrosine hydroxylase (TH) in the SNc. Behavioral assays, including balance beam walking, pole descent, grip strength, rotarod, and amphetamine-induced rotation, were used to assess for motor deficits. The involvement of CB2 receptors was further probed by testing whether the effects of GW842166x were blocked by the CB2 antagonist AM630. 

The selective vulnerability of the SNc dopamine neurons can be attributed to an interplay between high cytosolic dopamine, α-synuclein, and high cytosolic Ca^2+^ levels [28]. Dopamine release is triggered by action potential (AP) firing in dopamine neurons. SNc dopamine neurons exhibit autonomous pacemaker firing that produces a basal dopaminergic tone in their projection targets, in particular the striatum [29], and this basal dopamine tone is crucial for voluntary movement [30,31]. In adult SNc dopamine neurons, autonomous pacemaker firing is driven by the co-activation of hyperpolarization-activated cyclic nucleotide-gated (HCN) channels and L-type (Ca_v_1.3) Ca^2+^ channels [29,32], which leads to a Ca^2+^ influx [33]. CB2 agonists inhibit the action potential firing in ventral tegmental area (VTA) dopamine neurons [19,34]. Using ex vivo brain slice electrophysiology, we tested the hypothesis that GW842166x decreases the spontaneous activity of SNc dopamine neurons by reducing HCN activation. These results raise the possibility that the CB2 agonist GW842166x could be repurposed as a neuroprotective treatment in the early phase of PD.

## 2. Materials and Methods

### 2.1. Animals

C57BL/6J mice (Jax stock#: 000664) of either sex (10–12 weeks old) were purchased from The Jackson Laboratory (Bar Harbor, ME). All experiment groups had roughly equal numbers of male and female mice. Mice were given ad libitum access to food and water, unless stated otherwise, and housed four to five per cage in a temperature (23 ± 1 °C) and humidity-controlled room (40–60%) with a 14 h light, 10 h dark cycle. Animal maintenance and use were in accordance with protocols approved by the Institutional Animal Care and Use Committee of the Medical College of Wisconsin. 

### 2.2. Intra-Striatal 6-OHDA Injection

**Figure 1:**
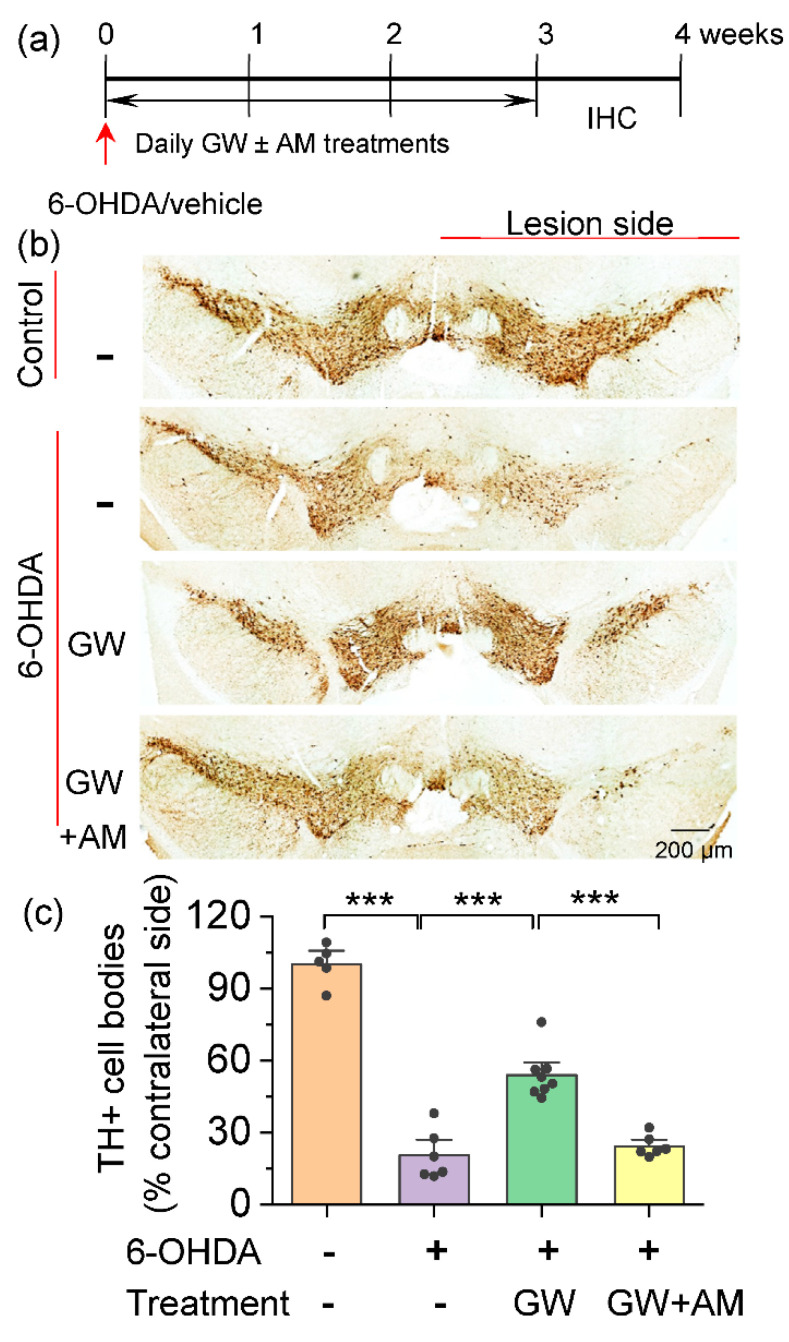
GW842166x (GW) protected against the 6-OHDA-induced loss of dopamine neurons. (**a**) Timeline of 6-OHDA or vehicle injection, GW/AM630 (AM) treatments and histology. (**b**) DAB staining for TH^+^ dopamine neurons in midbrain sections of mice that received intra-striatal injection of 6-OHDA or vehicle and chronic GW or GW + AM treatments. (**c**) Summarized data showing that the total number of TH^+^ dopamine neurons (% if contralateral side) in the SNc was significantly decreased in the 6-OHDA group relative to control (*** *p* < 0.001, 6 sections/mouse; n = 5–6 mice). Chronic GW treatments reduced 6-OHDA-induced dopamine neuron loss (*** *p* < 0.001, n = 6–8 mice), and this effect was blocked by co-treatment with AM630 (*** *p* < 0.001, n = 6–8).

Mice were anesthetized with ketamine (90 mg/kg, i.p.) and xylazine (10 mg/kg, i.p.) and placed in a robot stereotaxic system (Neurostar, Germany). A total volume of 2 µL 6-OHDA (2 µg/µL in PBS with 0.02% sodium L-ascorbate) or control vehicle (PBS and 0.02% sodium L-ascorbate) was delivered through a Nanoject III Programmable Nanoliter Injector (Drummond Scientific Company, Broomall, PA, USA). The stereotaxic coordinates for striatum injections were: anteroposterior, +0.5 mm; mediolateral, ±1.8 mm; dorsoventral, −3.0 and −2.0 mm (Paxinos and Franklin, 2001). The injection rate was 120 nL/min and the injectors were kept in place for 5 min to ensure adequate diffusion from the injector tip (~30 μm). Desipramine (Sigma; 25 mg/kg, i.p.) was injected 30 min prior to 6-OHDA injection to prevent the uptake of 6-OHDA by noradrenergic neurons [35]. After the surgery, animals received a subcutaneous injection of analgesic (buprenorphine-SR, 0.05 mg/kg). Mice began receiving drug treatments starting from the day after intra-striatal 6-OHDA or vehicle injection. The purpose of the study was to determine whether GW842166x was effective in reducing 6-OHDA-induced dopamine neuron loss and associated motor deficits, and whether the effect of GW842166x was mediated by CB2 receptors. Only controls essential to this goal were included to minimize the number of animals expended. The 4 essential groups consisted of mice that received intra-striatal microinjections of 6-OHDA or vehicle and then daily i.p. injections of saline, and 6-OHDA-injected mice that received daily i.p. injections of GW842166x (1 mg/kg) or GW842166x (1 mg/kg) + AM630 (10 mg/kg) for three weeks. After completion of drug treatments, immunohistochemical staining or behavioral tests were performed (see timeline in Figure 1 and Figure 4).

### 2.3. Immunohistochemistry (IHC)

Mice were anaesthetized by ketamine (90 mg/kg, i.p.) and xylazine (10 mg/kg, i.p.) and transcardially perfused with 0.1 M sodium PBS, followed by 4% paraformaldehyde in 4% sucrose PBS (pH 7.4). After perfusion, the brain was removed and post-fixed in the same fixative for 4 h at 4 °C and was then dehydrated by increasing concentrations of sucrose (20% and 30%) in 0.1 M PBS at 4 °C and frozen on dry ice. Coronal midbrain sections (25 µm) were cut with a CM1860 cryostat (Leica; Nussloch, Germany). The sections were incubated with primary antibodies against tyrosine hydroxylase (TH, rabbit, 1:300, Santa Cruz Biotechnology) at 4 °C for 48 h. After rinsing with PBS three times at 15 min each, midbrain sections were then incubated in secondary antibodies: anti-rabbit IgG HRP-conjugated (1:200, Jackson ImmunoResearch) for 4 h at room temperature. Immunoreactivity was visualized with 3,3′-Diaminobenzidine (DAB) Substrate Kit (SK-4100; Vector Laboratories, Inc., Burlingame, CA, USA) for 5 min, after which the reaction was stopped with PBS wash for 5 min and then rinsed in PBS, dehydrated, and cover-slipped. The sections were imaged with a Hamamatsu Slide Scanner and analyzed by ImageJ software. For dopamine neuron quantification, TH^+^ neurons in the SNc were counted from both brain hemispheres in 6 coronal sections from each mouse between approximately 3.1 and 3.6 mm posterior to bregma. The brain sections (25 μm) were sampled every third slice. Counts of dopamine neurons were expressed as the percentage of contralateral side. We compared the cell number differences within the same slices (control and lesion sides) and between different treatment groups, which minimized potential sampling errors.

### 2.4. Brain Slicing 

Adult drug-naïve mice of either sex were anesthetized by isoflurane inhalation and decapitated. The brain was removed, trimmed, and embedded in low-melting-point agarose, and horizontal slices (200 μm thick) containing the midbrain were cut using a vibrating slicer (Leica VT1200s), as described in our recent studies [36]. Slices were prepared in a cutting solution containing the following (in mM): 110 choline chloride, 2.5 KCl, 1.25 NaH_2_PO_4_, 0.5 CaCl_2_, 7 MgSO_4_, 26 NaHCO_3_, 25 glucose, 11.6 sodium ascorbate, and 3.1 sodium pyruvate. The midbrain slices were cut at the midline to produce two individual slices from each section. After slice cutting, ACSF was progressively spiked into the choline solution every 5 min for 20 min at room temperature to gradually reintroduce Na^+^, similar to a previous method [37]. The slices were allowed to recover for at least an additional 30 min in ACSF prior to recording. All solutions were continuously saturated with 95% O_2_ and 5% CO_2_.

### 2.5. Electrophysiology 

Whole-cell and cell-attached patch-clamp recordings were performed with patch-clamp amplifiers (Multiclamp 700B; Molecular Devices, San Jose, CA, USA) under infrared differential interference contrast (DIC) microscopy. Data were acquired using DigiData 1440A and 1550B digitizers and were analyzed with pClamp 10 (Molecular Devices). Signals were sampled at 10 kHz and filtered at 2 kHz. Recordings of action potential (AP) firing were created in the cell-attached mode, CNQX (10 µM), D-AP5 (20 µM) and picrotoxin (50 µM) were present in the ACSF to block synaptic currents. Junction potentials were nullified before obtaining a gigaohm seal. The I_h_ activation curves were generated by applying 1.5-s hyperpolarizing steps to various potentials (−60 to −130 mV) from a holding potential of −60 mV and tail currents were measured at −130 mV. Tetraethylammonium chloride (TEA-Cl, 10 mM) was present in the ACSF to block non-inactivating voltage-dependent K^+^ conductance. An equimolar reduction in NaCl from the ACSF maintained osmolality. The current amplitude following no hyperpolarizing step was subtracted from tail current amplitudes at −130 mV and plotted as a function of test potentials. The I_h_ activation curve was fitted with a Boltzmann function *I* = *I*_max_/exp((*V*_m_ − *V*_1/2_)/s), where *I*_max_ is the maximal tail current amplitude, *V* is the test potential, *V*_1/2_ is the half-activation potential, and *s* is the slope factor. Instantaneous inward currents (I_ins_) were generated by inducing 10 mV hyperpolarizing voltage steps from a resting holding potential of −60 mV to −130 mV. I_h_ amplitude was calculated by subtracting I_ins_ from the steady-state current (see Figure 3a). The amplitudes of evoked I_h_ were plotted against the hyperpolarizing voltage steps. Membrane capacitance was measured by pClamp 10 using −5 mV hyperpolarizing steps. Patch pipettes (3–5 MΩ) were filled with an internal solution containing (in mM): 140 K-gluconate, 10 KCl, 10 HEPES, 0.2 EGTA, 2 MgCl_2_, 4 Mg-ATP, 0.3 Na_2_GTP, 10 Na_2_-phosphocreatine (pH 7.2 with KOH). Series resistance (10–20 MΩ) was monitored throughout all recordings, and data were discarded if the resistance changed by more than 20%. An automatic temperature controller (Warner Instruments LLC, Hamden, CT, USA) was used to maintain the temperature for recordings at 32 ± 1 °C. 

### 2.6. Behavioral Tests 

One day after completion of three-week drug treatments, mice were subjected to different behavioral tests (see timeline in Figure 4a). 

#### 2.6.1. Pole Test

A pole test apparatus, consisting of a vertical pole (diameter 8 mm, height 50 cm) with a small piece of cardboard at the top to prevent mice from climbing over the pole, was placed into a home cage with soft bedding. Mice were placed individually on top of the pole with their head oriented upward and allowed to descend to the floor [38]. On the first day, mice received two training trials to learn how to descend the pole. On the second day, five pole descent trials were performed, and the lowest times required for animals to orient themselves in a downward direction (*T*_turn_) and to descend to the base of the pole (*T*_total_) were used for analysis. Trials were restarted if mice prematurely fell from the pole, and animals that slipped down the pole or failed to re-orient downwards were excluded from analysis.

#### 2.6.2. Balance Beam 

A custom-built wooden bar (100 cm length × 6 cm width) was placed 50 cm above the floor, with one end placed in a dark escape home cage. Testing was performed across three consecutive days. During the first two days, mice were habituated to the escape cage for 2 min, then placed at the starting point and trained to cross the balance beam to reach the escape cage three times. On the third day, mice were placed individually at the starting point and allowed to cross the balance beam. Each mouse was tested three times. The average time for mice to cross the beam and the number of foot slips on the testing day were analyzed. 

#### 2.6.3. Grip Strength

Grip strength of mice was tested using a DFE II Series Digital Force Gauges (AMETEK, Inc., Berwyn, PA, USA). The force gauge was positioned horizontally, and the mice were held by the tail and lowered towards the apparatus. The mice were allowed to grab the metal grid and were then pulled backwards in the horizontal plane. The force applied to the grid just prior to loss of grip was recorded as the peak grip strength. Each mouse was tested three times, and averaged grip strength was used for analysis [39,40].

#### 2.6.4. Accelerating Rotarod

The accelerating rotarod test was performed using a rotarod apparatus (IITC Life Science, Inc., Woodland Hills, CA, USA). The apparatus consisted of a computer-controlled motor-driven rotating spindle and five lanes for five mice. Mice that fell from the rotating spindle were detected automatically by a pressure plate at the base of the apparatus. Mice were habituated to the test environment for one hour prior to the test. For training, mice from the same cage were placed in separate lanes of the testing apparatus and the rod rotated at a constant speed of 5 rotations per minute (rpm) for 180 s. After the training, mice were tested in accelerating speed procedure in which the rotation speed accelerated from 4 to 40 rpm over the course of 5 min. The test was repeated for 3 days, with 3 trials each day, separated by 15 min inter-trial intervals. The mean latency to fall from the rotating rod across trials was recorded each day.

#### 2.6.5. Spontaneous and Amphetamine-Induced Rotation

The amphetamine-induced rotation test [41] was performed using glass cylinders (ID = 15 cm, height = 20 cm). Mice were first placed individually into a glass cylinder and spontaneous full body rotations in the direction ipsilateral (+) or contralateral (−) to the injected hemisphere were recorded for 40 min. Then, mice were injected with d-amphetamine (5 mg/kg, i.p.) and put back into the same cylinder. Ten minutes after d-amphetamine injection, rotations were recorded for another 40 min. Full body rotations in the ipsilateral and contralateral directions were counted in 5 min intervals. The net rotations (ipsilateral rotations–contralateral rotations) before and after d-amphetamine injection were reported. 

### 2.7. Chemicals

The 6-OHDA was purchased from Sigma-Aldrich (St. Louis, MO, USA). D-amphetamine was provided by the NIDA Drug Supply Program. GW842166X and AM630 were purchased from Cayman Chemical Company (Ann Arbor, MI, USA). GW842166X and AM630 were first dissolved in DMSO (Sigma-Aldrich) and then mixed with TWEEN-80 (Sigma-Aldrich). Then, sterile saline was added to the mix solution to create a final working solution with 2.5% DMSO + 2.5% TWEEN-80 + 95% saline. Picrotoxin and all other common chemicals were obtained from Sigma-Aldrich. 6-Cyano-7-nitroquinoxaline-2,3-dione disodium salt (CNQX) and D-(-)-2-Amino-5-phosphonopentanoic acid (D-AP5) were obtained from Tocris Bioscience (Ellisville, MO, USA). 

### 2.8. Statistics 

Data were presented as the mean ± SEM. Data sets were compared with either Student’s *t*-test, one-way or two-way ANOVA, or repeated measures ANOVA, followed by Tukey’s post hoc analysis. Post hoc analyses were performed only when ANOVA yielded a significant main effect or a significant interaction between the two factors. Results were considered to be significant at *p* < 0.05.

## 3. Results

### 3.1. CB2 Agonism Protected Dopamine Neurons against Degeneration Induced by 6-OHDA 

We examined whether the CB2-selective agonist GW842166x protected against dopamine neuron loss in the SNc. The 6-OHDA injection resulted in a significant loss of dopamine neurons within two weeks [42]. C57BL/6J mice received a single unilateral injection of 6-OHDA or control vehicle at two sites in the striatum. Immediately after the surgery, mice began receiving the vehicle, GW842166x (1 mg/kg), or GW842166x (1 mg/kg) + AM630 (10 mg/kg) treatments daily for three weeks. The doses of GW842166x and AM630 were based on previous studies with adjustment for animal species [43,44]. The day after the last treatment, an immunohistochemical staining for tyrosine hydroxylase (TH^+^) of the midbrain sections was performed (Figure 1a). The one-way ANOVA indicated significant main effects of GW842166x treatment and 6-OHDA injection on the number of TH^+^ somata (*F*_1,24_ = 95.0, *p* < 0.001; Figure 1b,c). Tukey’s post hoc tests indicated that the 6-OHDA injection significantly reduced the number of TH^+^ dopamine neurons in the SNc compared with the vehicle injection (*p* < 0.001; Figure 1c). Chronic treatment with GW842166x significantly reduced the loss of TH^+^ dopamine neurons in the SNc induced by 6-OHDA (*p* < 0.001; Figure 1c), and these effects were blocked by co-treatment with AM630 (*p* < 0.001; Figure 1c). These results suggest that GW842166x protects against the neurotoxic effects of 6-OHDA on dopamine neurons, and this protective effect is mediated by the activation of CB2 receptors.

### 3.2. Mechanisms by Which the CB2 Agonist GW842166x Exerts Neuroprotective Effects

**Figure 2:**
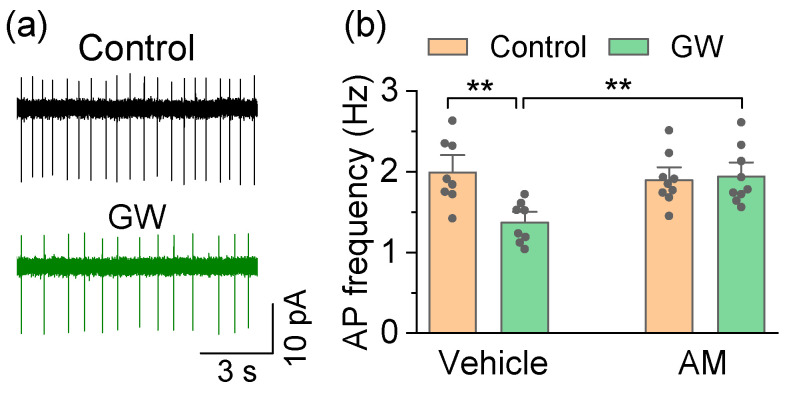
GW842166x (GW) decreased autonomous AP firing in SNc dopamine neurons in drug-naïve mice. (**a**) Bath application of GW (1 µM) decreased AP firing in SNc dopamine neurons. (**b**) Summarized data demonstrating that bath application of GW decreased the frequency of AP firing in SNc dopamine neurons (** *p* = 0.004, n = 8 cells from 3 mice), and this decrease was blocked by pre-incubation of the slice with AM630 (AM) (** *p* = 0.008, n = 8–9 cells from 3 mice).

The neurodegeneration in PD occurs in SNc dopamine neurons, but the neighboring dopamine neurons in the VTA are largely spared [45]. The selective venerability of SNc dopamine neurons can be attributed to an interplay between high cytosolic dopamine, α-synuclein, and high cytosol Ca^2+^ levels [28]. Dopamine release is triggered by action potential (AP) firing. SNc/VTA dopamine neurons exhibit autonomous pacemaker firing that is necessary to maintain a basal dopamine tone in their projection targets, including the striatum [29]. In adult SNc dopamine neurons, the autonomous pacemaker firing is driven by the co-activation of hyperpolarization-activated cyclic nucleotide-gated channels (HCN) and L-type (Ca_v_1.3) Ca^2+^ channels [29,32], leading to a sustained Ca^2+^ entry and mitochondrial stress [33]. Compared to SNc dopamine neurons, VTA dopamine neurons express a much lower Ca_v_1.3 Ca^2+^ channel density [46], high levels of the Ca^2+^-buffering protein calbindin [47], and have a significantly lower risk of degeneration in PD [47,48]. The CB2 receptor mRNA and protein are expressed by midbrain dopamine neurons, and CB2 agonists inhibit pacemaker AP firing in VTA dopamine neurons [19], but their effects on AP firing in SNc dopamine neurons have not been examined. We investigated whether GW842166x altered autonomous pacemaker activity in SNc dopamine neurons. Midbrain slices were prepared from adult (10–12 weeks old) drug-naïve C57BL/6J mice. Non-invasive, tight-seal cell-attached patch-clamp recordings were obtained from SNc dopamine neurons, which could be readily identified by their location and characteristic firing pattern. The recordings were performed in the presence of the AMPA receptor blocker CNQX (10 µM), NMDA receptor blocker D-AP5 (20 µM), and GABA_A_ receptor blocker picrotoxin (50 µM) to block the synaptic transmission. Stable baseline AP firing was first established prior to drug administration. GW842166x (1 µM) or the vehicle was perfused to test their effect on AP firing. To determine the involvement of the CB2 receptor, we also performed these recordings in slices pre-incubated with AM630 (10 µM). The two-way ANOVA found that GW842166x and AM630 had a significant main effect on the AP firing frequency in SNc dopamine neurons (GW842166x: *F*_1,33_ = 6.4, *p* = 0.017; AM630: *F*_1,33_ = 4.2, *p* = 0.048; GW842166x × AM630 interaction: *F*_1,33_ = 8.4, *p* = 0.007; Figure 2a,b). Tukey’s post hoc tests revealed that GW842166x application significantly decreased the AP firing frequency in SNc dopamine neurons (*p* = 0.004; Figure 2a,b), and this decrease did not occur in neurons from slices preincubated with AM630 (*p* = 0.008; Figure 2a,b).

There are several potential mechanisms that may explain the GW842166x-induced decrease in autonomous AP firing in SNc dopamine neurons. The CB2 receptor is a G_i/o_-coupled G-protein-coupled receptor (GPCR) that leads to a decrease in cAMP [49]. The two pacemaker channels of SNc dopamine neurons, Ca_v_1.3 and HCN, are sensitive to cAMP [50,51], and a sustained decrease in cAMP would decrease the activation of both channels. However, Ca_v_1.3 is difficult to isolate pharmacologically, so we focused our studies on whether GW842166x may alter I_h_. HCN channels are gated by cAMP and activated by hyperpolarization [51]. Of the four HCN channel subtypes, HCN2 and HCN4 are sensitive to cAMP [52]. SNc dopamine neurons express HCN2-4, as shown by the single-cell RT-PCR and in situ hybridization, and the expression of the cAMP-sensitive subtypes (HCN2 and HCN4) is predominant [53,54]. cAMP binding to the cyclic nucleotide-binding domain of HCN2 produced a marked depolarizing shift (up to 17 mV) in half-activation potential (*V*_1/2_) that greatly facilitated voltage-dependent activation [52,55]. A CB2-dependent decrease in cAMP concentrations was expected to shift the *V*_1/2_ of HCN to a more hyperpolarized potential, dampening the voltage-dependent activation. 

**Figure 3:**
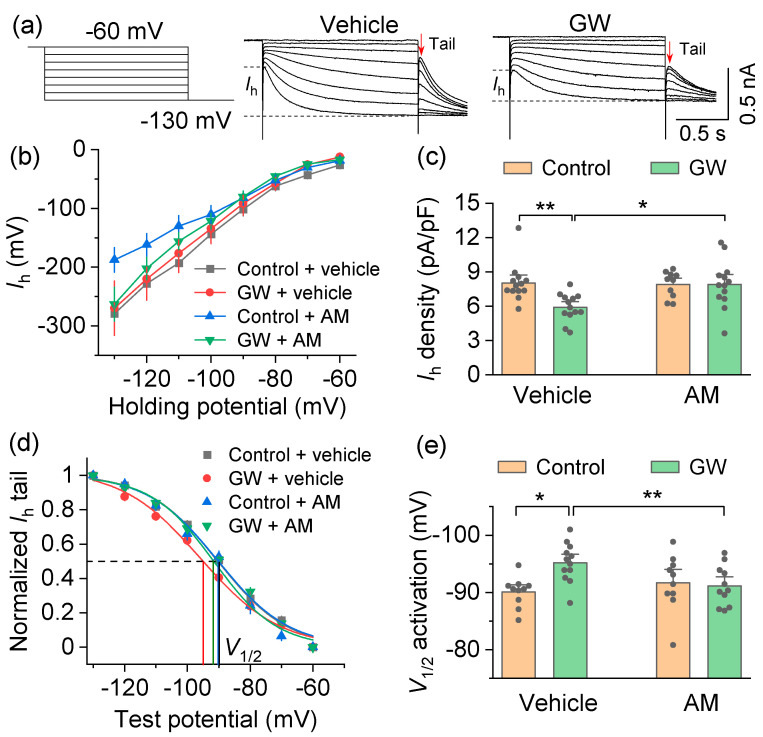
Activation of the CB2 receptor decreased HCN currents (I_h_) in SNc dopamine neurons. (**a**) Left, A hyperpolarizing voltage step protocol used to induce I_h_. Right, representative I_h_ traces from SNc dopamine neurons following bath application of GW842166x (GW) or control. (**b**) I–V relationship of I_h_ recorded from different treatment groups. (**c**) I_h_ density was significantly decreased by bath application of GW compared to vehicle (** *p* = 0.007, n = 13 cells from 4 mice), and the GW-induced decrease in I_h_ density was blocked by AM pretreatment (* *p* = 0.012, n = 13 cells from 4 mice). (**d**) I_h_ activation curves generated by the tail current protocol in SNc dopamine neurons. (**e**) Bath application of GW led to a hyperpolarizing shift in the half-activation potential (*V*_1/2_) compared with control (* *p* = 0.011, n = 10–12 cells from 4 mice), and this shift was prevented by AM preincubation (** *p* = 0.001, n = 10–11 cells from 4 mice).

The I_h_ current was induced by hyperpolarizing voltage steps (from −60 mV to −130 mV with −10 mV steps, 1.5 sec duration), followed by a step to −130 mV for the analysis of tail currents (Figure 3a,b). We next normalized the I_h_ amplitude at −130 mV to the cell capacitance and to determine the I_h_ density (Figure 3c). The cell capacitance (C_m_) was monitored throughout the recordings and no significant changes were detected by drug treatments (GW842166x: *F*_1,48_ = 0.3, *p* = 0.570; AM630: *F*_1,48_ = 0.007, *p* = 0.932; GW842166x x AM630 interaction: *F*_1,48_ = 0.3, *p* = 0.594). The two-way ANOVA found that GW842166x and AM630 had significant main effects on the I_h_ density (GW842166x: *F*_1,48_ = 5.4, *p* = 0.025; AM630: *F*_1,48_ = 4.3, *p* = 0.045; GW842166x × AM630 interaction: *F*_1,48_ = 5.4, *p* = 0.025; Figure 3c). Tukey’s post hoc tests revealed that the GW842166x application significantly decreased the I_h_ density in SNc dopamine neurons (*p* = 0.007), and this decrease was reversed by preincubating slices with AM630 (*p* = 0.012; Figure 3c). To examine whether GW842166x altered the I_h_ activation properties, we plotted the tail current amplitudes as a function of test potentials and fit them to a Boltzmann function to produce I_h_ activation curves (Figure 3a,d). The two-way ANOVA found that GW842166x and AM630 had significant main effects on the *V*_1/2_ of SNc dopamine neurons (GW842166x: *F*_1,42_ = 5.1, *p* = 0.030; AM630: *F*_1,42_ = 10.2, *p* = 0.003; GW842166x × AM630 interaction: *F*_1,42_ = 5.6, *p* = 0.022; Figure 3e). Tukey’s post hoc tests revealed that the GW842166x application significantly shifted the *V*_1/2_ to a more hyperpolarized potential (*p* = 0.010), and this shift was prevented by preincubation with AM630 (*p* = 0.001; Figure 3e). These results indicated that the CB2 activation decreased HCN channel-mediated pacemaker currents and increased the level of hyperpolarization necessary to activate these currents in SNc dopamine neurons. 

### 3.3. GW842166x Protected against 6-OHDA-Induced Motor Functions Deficits

**Figure 4:**
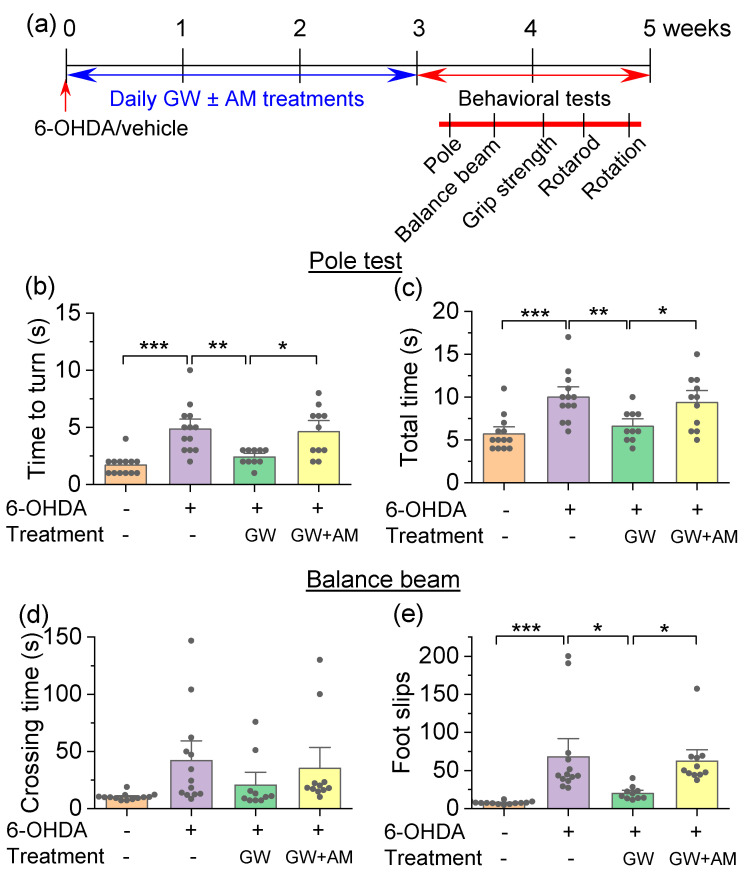
GW842166x (GW) treatment attenuated 6-OHDA-induced motor deficits in the pole test and balance beam test. (**a**) Timeline of 6-OHDA injection, drug treatments, and behavioral tests. (**b**,**c**) In the pole test, mice injected with 6-OHDA took more time to reorient downwards (*T*_turn_; *** *p* < 0.001, n = 13–13) and to descend the pole (*T*_total_; *** *p* < 0.001, n = 13–13) compared with control mice. GW treatment prevented the prolongation of *T*_turn_ (** *p* = 0.004, n = 10–13) and *T*_total_ (** *p* = 0.007, n = 10–13) induced by 6-OHDA injection. The protective effects of GW were prevented by co-treatment with AM630 (AM) (*T*_turn_, * *p* = 0.014; *T*_total_, * *p* = 0.044, n = 10–11). (**d**) In the balance beam test, the average time to cross the beam was not significantly affected by 6-OHDA injection, chronic GW treatment, or co-treatment with GW and AM (*p* > 0.05, n = 10–13). (**e**) The number of foot slips from the beam was significantly increased in the 6-OHDA group relative to control (*** *p* < 0.001, n = 13–13), the increase in foot slips was attenuated by GW treatments (* *p* = 0.011, n = 10–13), and AM co-treatments prevented the effect of GW (* *p* = 0.039, n = 10–11).

We next determined the extent to which GW842166x protected against 6-OHDA-induced motor function deficits. The 6-OHDA injection and drug treatments were the same as described in Figure 1, except that the behavioral experiments were carried out starting one day after the last drug treatments. The four groups of mice were subject to behavioral tests, including the pole test, balance beam, grip strength, rotarod tests, and amphetamine-induced rotation assays (Figure 4a). The pole test evaluated the ability of a mouse to grasp, maneuver, and descend a vertical pole in its home cage [38]. Mice were placed individually at the top of the pole with their head facing upward, and the latency required to re-orient facing downward (*T*_turn_) and then descend the pole (*T*_total_) was recorded. One-way ANOVA indicated that drug treatment had a significant main effect on the time needed for mice to turn (*T*_turn_; *F*_3,43_ = 11.8, *p* < 0.001; Figure 4b) and the total time to descend the pole (*T*_total_; *F*_3,43_ = 9.0, *p* < 0.001; Figure 4c). Tukey’s post hoc tests revealed that 6-ODHA prolonged *T*_turn_ (*p* < 0.001) and *T*_total_ (*p* < 0.001), indicating motor dysfunction. The GW842166x treatment decreased *T*_turn_ (*p* = 0.004) and *T*_total_ (*p* = 0.007) in 6-OHDA-exposed mice. The protective effects of GW842166x were prevented by co-treatment with AM630 (*T*_turn_, *p* = 0.014; *T*_total_, *p* = 0.044; Figure 4b,c). 

The balance beam walking assay analyzed the motor coordination of the fore- and hindlimbs and was sensitive to unilateral 6-OHDA lesions in mice [56]. In this assay, mice had to maintain balance while crossing a narrow beam. The one-way ANOVA revealed no significant main effect of drug treatment on the time needed for mice to traverse the balance beam (*F*_3,43_ = 2.1, *p* = 0.111; Figure 4d). However, there was a significant main effect of drug treatment on the number of foot slips during beam crossing (*F*_3,43_ = 9.1, *p* < 0.001; Figure 4e). Tukey’s post hoc tests revealed that 6-OHDA-injected mice experienced significantly more foot slips compared with control mice (*p* < 0.001; Figure 4e), and this increase was attenuated by GX842166x treatment (*p* = 0.011; Figure 4e). The effect of GW842166x was prevented by co-treatment with AM630 (*p* = 0.039; Figure 4e).

**Figure 5:**
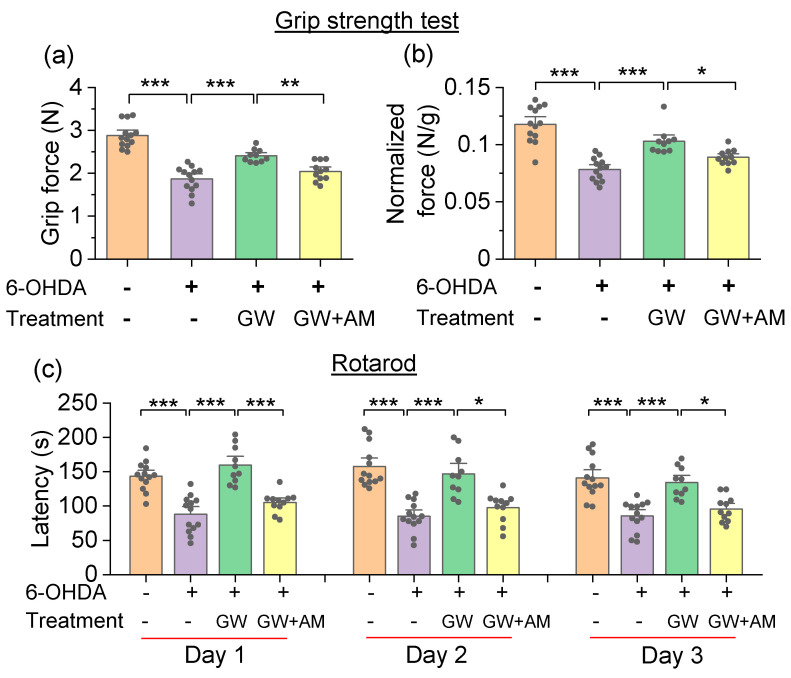
GW842166x (GW) treatment attenuated 6-OHDA-induced motor deficits in the grip strength test and rotarod test. (**a**,**b**) In the grip strength test, 6-OHDA injection decreased the absolute grip strength (grip force, *** *p* < 0.001, n = 13–13) and normalized grip strength (normalized force, *** *p* < 0.001, n = 13–13), both of which were ameliorated by GW treatment (*** *p* < 0.001, n = 10–13). Co-treatment with AM630 (AM) prevented the protective effects of GW (** *p* = 0.009, * *p* = 0.044, n = 10–11). (**c**) In the rotarod test, 6-OHDA injection significantly decreased the latency to fall (*** *p* < 0.001, n = 13–13), GW treatments significantly increased the latency to fall (*** *p* < 0.001, n = 10–13), and AM co-treatments prevented the effect of GW (* *p* < 0.05, *** *p* < 0.001, n = 10–11).

The grip strength test quantified muscular strength [39,40]. To account for differences in grip strength due to animal size, grip strength was normalized to body weight for each mouse. In the grip strength test, there was a significant main effect of drug treatment in the absolute grip strength (*F*_3,43_ = 40.9, *p* < 0.001; Figure 5a) and normalized grip strength (*F*_3,43_ = 27.0, *p* < 0.001; Figure 5b). Tukey’s post hoc tests revealed that 6-ODHA injection decreased the absolute grip strength (*p* < 0.001; Figure 5a) and normalized grip strength (*p* < 0.001; Figure 5b) relative to control mice, both of which were ameliorated by the GW842166x treatment (*p* < 0.001; Figure 5a,b). The effect of GW842166x was prevented by co-treatment with AM630 (*p* = 0.009; Figure 5a,b).

In the rotarod test, mice were placed on a horizontal rod that rotated with an accelerating velocity. Mice had to maintain balance and an upright position while engaging in forward locomotion to avoid falling. The experiments were performed daily for three days. The two-way repeated-measures ANOVA revealed that there was a significant main effect of drug treatment on the latency to fall (*F*_3,43_ = 38.2, *p* < 0.001). There was no significant main effect of testing day on the latency to fall (*F*_2,86_ = 3.2, *p* = 0.054), and there was no significant interaction between the testing day and drug treatment (*F*_6,86_ = 1.5, *p* = 0.200; Figure 5c). An a priori comparison indicated that the 6-OHDA injection significantly decreased the latency to fall relative to control mice (day 1, *p* < 0.001; day 2, *p* < 0.001; day 3, *p* < 0.001). The GW842166x treatment in 6-OHDA-exposed mice significantly increased the latency to fall (day 1, *p* < 0.001; day 2, *p* < 0.001; day 3, *p* < 0.001), and the latency to fall did not differ between these mice and control mice (*p* > 0.05). The effect of GW842166x was prevented by co-treatment with AM630 (day 1, *p* < 0.001; day 2, *p* < 0.001; day 3, *p* = 0.015; Figure 5c).

**Figure 6:**
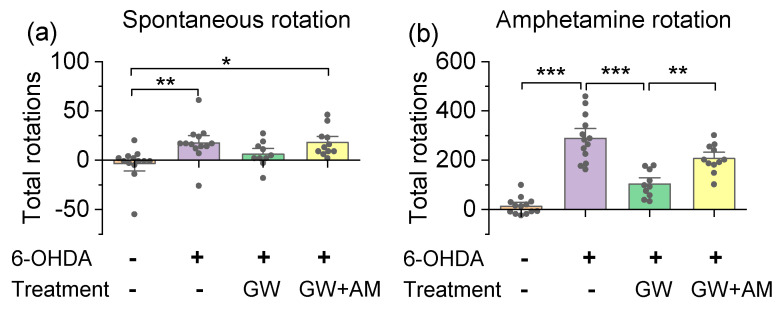
GW842166x (GW) treatment decreased spontaneous and amphetamine-induced rotations. (**a**) Mice unilaterally injected with 6-OHDA exhibited an increase in spontaneous rotations compared to vehicle-injected mice (** *p* = 0.009, n = 13–13). GW without AM630 (AM) co-treatment did not significantly alter the number of spontaneous rotations relative to control mice (*p* = 0.484, n = 10–13). GW plus AM co-treatment significantly increased the number of spontaneous rotations relative to 6-OHDA controls (* *p* = 0.011, n = 11–13). (**b**) Unilateral 6-OHDA injection increased amphetamine-induced (5 mg/kg, i.p.) net rotations relative to control mice (*** *p* < 0.001, n = 13–13). This increase in rotations was attenuated by GW treatment (*** *p* < 0.001, n = 10–13), and the effect of GW was prevented by AM co-treatment (*** *p* = 0.004, n = 10–11).

We also investigated the protective effects of GW842166x in both the spontaneous and amphetamine-induced rotation assays [41]. A unilateral intra-striatal injection of 6-OHDA degenerated dopamine neurons in the ipsilateral SNc, while dopamine neurons in the contralateral hemisphere were unaffected. This resulted in an imbalance of dopamine release between the hemispheres and an asymmetrical movement. Mice were placed individually into glass cylinders and spontaneous rotations in both directions were recorded for 40 min. The one-way ANOVA indicated a significant main effect of drug treatment on spontaneous rotations (*F*_3,43_ = 5.1, *p* = 0.004, Figure 6a,b), and the Tukey’s post hoc analysis showed that the 6-OHDA group exhibited a significant increase in the net spontaneous rotations in the direction ipsilateral to the injection site compared to the control group (*p* = 0.009; Figure 6a). Next, mice received an i.p. injection of d-amphetamine (5 mg/kg, i.p.), and rotations were recorded for another 40 min. The one-way ANOVA showed that there was a significant effect of drug treatment on d-amphetamine-induced rotations (*F*_3,43_ = 43.8, *p* < 0.001; Figure 6b). The 6-OHDA-lesioned mice performed more net rotations in the direction ipsilateral to the injection site compared to control mice (*p* < 0.001). The increase in rotations was attenuated by the GW842166x treatment (*p* < 0.001), and the effect of GW8421266x was prevented by the AM630 co-treatment (*p* = 0.004; Figure 6b). Taken together, the above results indicated that 6-OHDA impaired motor function in mice in a variety of behavioral tests, and that GW842166x ameliorated the 6-OHDA-induced motor function deficits in a CB2-dependent manner. 

## 4. Discussion

The etiology of PD is heterogenous, with both genetic and environmental determinants [2]. The fact that there is no single cause of PD in humans is reflected in the wide range of neurotoxic and genetic animal models of PD used in preclinical research [57]. Importantly, there is increasing evidence that CB2-selective agonists are neuroprotective across multiple PD models. Β-caryophyllene, AM1241, and HU-308 were shown to be protective against dopaminergic neurotoxicity induced by rotenone [23], MPTP [58], and LPS [59], respectively. We extended these studies by demonstrating the neuroprotective effects of a novel CB2-selective agonist, GW842166x, against 6-OHDA-induced neurodegeneration, as well as motor function deficits. We investigated the mechanisms involved and found that GW842166x decreased the action potential firing of SNc dopamine neurons by reducing the activation of HCN channel-mediated currents. It is likely that the CB2 agonist protected against dopamine neuron degeneration by reducing AP firing and the associated calcium influx/load.

The 6-OHDA could be unilaterally injected into the SNc, medial forebrain bundle (MFB), or dorsal striatum to induce the degeneration of dopamine neuron cell bodies in the SNc and dopaminergic nerve terminals in the striatum [60]. We targeted the dorsal striatum because the model produced a selective lesion of dopamine neurons in the SNc, while VTA dopamine neurons were largely spared (Figure 1), and the partial lesion of the midbrain dopamine neurons resembled the earlier stages of PD [60], when neuroprotective effects would be most impactful. A single 6-OHDA injection resulted in the loss of TH^+^ cell bodies in the SNc ipsilateral to the 6-OHDA injection as determined by TH immunohistochemistry. Treatments with the CB2-selective agonist GW842166x for three weeks attenuated the 6-OHDA-induced dopamine neuron loss, and this effect was prevented by the selective CB2 antagonist AM630. Thus, GW842166x protected against dopamine neurons loss via CB2 receptor-dependent mechanisms.

What might be the mechanisms for CB2-induced neuroprotection? The CB2 receptor mRNA and protein are expressed in midbrain dopamine neurons [19,20,21]. CB2 agonists inhibit pacemaker AP firing in VTA dopamine neurons [19,34], and the enhancement of M-type K^+^ channel activation contributes to the CB2-mediated suppression of AP firing in these neurons [34]. However, whether CB2 agonists alter AP firing in SNc dopamine neurons has not been examined. We showed that GW842166x inhibited spontaneous AP firing in SNc dopamine neurons, which was blocked by AM630, indicating CB2-dependent mechanisms. The CB2 receptor is a G_i/o_-coupled GPCR that leads to a decrease in cAMP [49]. The pacemaker channel HCN contributes to the pacemaking activity of SNc dopamine neuron [32,61], although Ca_v_1.3 Ca^2+^ channels may also participate [29]. Both HCN and Ca_v_1.3 are sensitive to cAMP [50,51], and a CB2-mediated decrease in cAMP would decrease the activation of these channels. We found that the bath application of GW842166x decreased the *V*_1/2_ and I_h_ amplitude in SNc dopamine neurons, which were blocked by AM630. The GW842166x-induced decrease in the *V*_1/2_ would shift the threshold of the HCN channel activation to a more hyperpolarized potential, resulting in a requirement for a greater hyperpolarization to activate HCN channels and engage the spontaneous AP firing. 

Why would the inhibition of SNc dopamine neuron AP firing provide neuroprotection? Ca^2+^ overload is a primary reason as to why SNc dopamine neurons are vulnerable to neurodegeneration [28]. The 6-OHDA is known to result in significant oxidative stress to dopamine neurons, which ultimately results in Ca^2+^-mediated cytotoxicity [62]. GW842166x may reduce the vulnerability of dopamine neurons to 6-OHDA by reducing the spontaneous action potential firing of these neurons and the associated calcium influx. However, CB2 receptors in non-neuronal cells may also confer neuroprotective effects of GW842166x. The injection of 6-OHDA or lipopolysaccharide (LPS) into the rat striatum led to an increased expression of the CB2 receptor as assessed by a real-time quantitative reverse transcription PCR, and this increase correlated significantly with an increase in microglial activation [63]. The CB2 gene expression was also significantly increased in the substantia nigra in the postmortem brains of patients with PD, and immunohistochemical analyses revealed that CB2 co-localized with astrocytes but not with neurons or microglia [22]. It is possible that both neuronal and non-neuronal mechanisms confer CB2-mediated neuroprotection in PD.

A critical question is whether GW842166x prevents the development of motor deficits in the 6-OHDA model of PD. We carried out an array of behavioral tests to assess motor function, including the pole test, balance beam, grip strength, rotarod, and amphetamine-induced rotation assays. Our results consistently demonstrated that treatment with the selective CB2 agonist significantly reduced 6-OHDA-induced motor deficits. Bradykinesia, a slowness of movement resulting from an impaired voluntary motor control, is one of the primary manifestations of PD [3], and was observed in increased latencies to complete tasks involving locomotion and balance. Fine motor coordination and balance were assessed with the pole test, balance beam, and rotarod tests. In the pole test, we found that the 6-OHDA lesion led to increases in both the time necessary for mice to turn and the time to descend the pole. The 6-OHDA group demonstrated greater difficulty traversing the balance beam and had more foot slips compared with the control group, indicating that balance and coordination were impaired. Similarly, in the rotarod test, mice in the 6-OHDA group had shorter latencies to fall from the rotating rod, adding further evidence for motor impairment resulting from the loss of SNc dopamine neurons. In the grip strength test, the 6-OHDA group showed a significant decrease in the limb muscle strength. The 6-OHDA-induced unilateral nigrostriatal lesion also resulted in asymmetric motor impairments as shown by increased spontaneous and amphetamine-induced rotations ipsilateral to the lesion side. The effects of the 6-OHDA lesion across the range of behavioral measures tested were all prevented or attenuated following three weeks of GW842166x treatment in a CB2-dependent manner. This indicated that CB2 activation is neuroprotective against 6-OHDA-induced motor deficits. 

## 5. Conclusions

In summary, we demonstrated that GW842166x, a CB2-selective agonist, attenuated the 6-OHDA-induced degeneration of dopamine neurons in the SNc and its associated motor deficits. GW842166x-induced protective effects were prevented by co-treatments with the selective CB2 antagonist AM630. Our study lends further support for the therapeutic potential of selective CB2 agonists against the degenerative effects of neurotoxic molecules with varying mechanisms of action. Although non-selective CB receptor agonists have shown neuroprotective effects against PD [14], targeting the CB1 receptor selectively to ameliorate symptoms of Parkinson’s disease has yielded inconsistent results [64]. Furthermore, the psychoactive effects of CB1 agonists may pose a risk for abuse [18]. As GW842166x was found to be safe and well-tolerated with no serious adverse effects in clinical trials [27], our study raises the exciting possibility that GW842166x or other CB2 agonists may be utilized as a neuroprotective treatment during the early phase of PD to slow disease progression. 

## Data Availability

The data presented in this study are available on request from the corresponding author.
